# The impact of the COVID-19 pandemic on lifestyle behaviors in children and adolescents: an international overview

**DOI:** 10.1186/s13052-022-01211-y

**Published:** 2022-02-04

**Authors:** S. Scapaticci, C. R. Neri, G. L. Marseglia, A. Staiano, F. Chiarelli, E. Verduci

**Affiliations:** 1 Department of Paediatrics, University of Chieti-Pescara, Chieti, Italy; 2 Department of Paediatrics, University of Pavia IRCCS San Matteo foundation, Pavia, Italy; 3 Department of Paediatrics, University of Naples “Federico II”, Naples, Italy; 4 Department of Paediatrics, Children’s Hospital “Vittore Buzzi”, University of Milan, Milan, Italy

**Keywords:** COVID-19 and children, SARS-CoV-2 infection and children, COVID-19 and nutrition, COVID-19 and lifestyle

## Abstract

The adverse effects of Severe Acute Respiratory Syndrome Coronavirus 2 (SARS-CoV-2) are not limited to the related infectious disease. In children and adolescents, serious risks due to the coronavirus disease 2019 (COVID-19) pandemic are also related to its indirect effects. These include an unbalanced diet with an increased risk of weight excess or nutritional deficiencies, increased sedentary lifestyle, lack of schooling, social isolation, and impaired mental health.

Pediatricians should be aware of the side effects of the COVID-19 pandemic on children’s diet, physical mental health and advise the families according to their nutritional needs and financial resources. Moreover, the lack of a targeted therapy able to offer protection against the deleterious effects of SARS-CoV-2 infection should require a greater effort by scientific societies to find a more effective prevention strategy. In this context, much interest should be given to nutritional support, able to contrast malnutrition and to stimulate the immune system.

## Background

Starting in 2020, the COVID-19 pandemic, whose causative agent is SARS-CoV-2, has represented the primary global issue influencing lifestyle behaviors of people worldwide. Wuhan, in China, has been the first city in which the SARS-CoV-2 virus has been detected, but soon the virus dissemination spread all over the world forcing World Health Organization (WHO) to declare the global pandemic state on 11th March 2020 [1]. SARS-CoV-2 is a pathogen with a particular tropism for respiratory tract epithelial cells leading to respiratory clinical pictures of various degrees of gravity, ranging from mild form characterized by cough, sore throat, and rhinitis to respiratory failure and acute respiratory distress (ARDS), secondary to virus colonization of the most distal region of the bronchial tree [2]. Respiratory symptoms frequently are associated with fever, headache, asthenia, diarrhea, vomiting, anosmia and, ageusia [3]. In severe cases of COVID-19, the viral dissemination into the bloodstream may determine secondary involvement of kidneys, esophagus, bladder, ileum, heart tissues, and central nervous system [4].

COVID-19 affects the pediatric population with a prevalence of 5% of all the cases diagnosed worldwide, with predominance for children and adolescents exposed to infected family members [5, 6]. Accordingly to data collected from different reports of the Literature, the median age at the diagnosis is of 6.6 years [7]. A recent meta-analysis reported a higher prevalence in female cases than males (56%) [2]. Fortunately, children seem to have a milder clinical course of COVID-19 than adults [1]. A large-scale study conducted on more than 2000 pediatric patients in China, observed that the rate of asymptomatic children with confirmed/suspected COVID-19 was 94.1% of whose 4.4% were completely asymptomatic while approximately 6% developed severe disease [5]. This may explain the observed low mortality of < 1% of all deaths [8].

The most frequent symptoms in the pediatric patients affected with COVID-19 are fever (57% with CI 49.7–64%) and cough (44.1 with CI 38.3–50.2%) [9]. However, influenza-like symptoms (headache, fatigue, rash, myalgia, dyspnea, rhinorrhea, nasal congestion, sore throat, nausea, vomiting, and abdominal pain) often coexist, making difficult to formulate a differential diagnosis [6, 10]. In addition, olfactory and gustatory abnormalities (typical of adult cases of SARS-CoV-2 infection) are rare in pediatric populations [11, 12]. The reasons why children and adolescents with SARS-CoV-2 infection have a milder disease course than adults are not yet clear. It has been suggested that biological factors in children, such as an immature immune system, could play a role to offer protection against damaging effects secondary to viral infection and abnormal immune system activation [13]. Moreover, differences in angiotensin-converting enzyme 2 (ACE2) expression may explain why SARS-CoV-2 infection is milder among children than adults as it represents the main gate for infection. The interaction between the SARS-CoV-2 envelope-anchored spike protein and ACE2 allows the entry of the virus into the type II alveolar cells of the lung. As a protective factor against SARS-CoV-2 infection, children seem to have a different ACE2 configuration, concentration, or binding capacity or a less harmful alveolar epithelial cell response to ACE2 when compared with adults [14].

However, the possibility to evolve towards severe disease stages is rare, but not impossible. In a pediatric case series of children with COVID-19, 30.8% presented dyspnea requiring ventilator support with oxygen, while 23.1% developed organ dysfunction, in the form of Multisystem Inflammatory Syndrome in Children (MIS-C), requiring intensive care unit (ICU) [15]. Recent epidemiological data reported the possibility to detect ground-glass opacities, a typical radiological finding of adult patients affected with COVID-19, in 35.5% (CI 28.9–42.7%) of all diagnosed pediatric cases, while the hospitalization rate seems to settle to 96.3% (CI 92.4–98.2%) of which 10.8% (CI 4.2–25.3%) with ICU admissions, and a rate of mortality of 2.4% (CI 1.7–3.4%) [16].

Given the high prevalence of asymptomatic cases and the lack of a single consensus about the screening procedure, the possibility to detect a large number of pediatric subjects affected with SARS-CoV-2 infection is reduced [17]. Add to this, the rate of newly reported COVID-19 cases depends on the local testing strategy, laboratory capacity, and the effectiveness of surveillance systems [18].

As a particular feature, pediatric patients with mild/moderate COVID-19 are likely to have higher nasal viral loads responsible for a silent spread in communities like schools, children’s playrooms [19] and mostly it might offer the chance to infect grandparents and other family members who are at risk to develop a severe course of the disease. However, their role in transmitting SARS-CoV-2 infection could be underestimated.

Given the high rate of SARS-CoV-2 contagiousness and rapid diffusion, since March 2020, there have been introduced multiple restrictions on human activities and physical interactions worldwide to prevent the spread of the virus, which forced people to stay at home influencing their food habits and lifestyles with potentially negative health consequences [20].

One of the first decisions taken by states and local governments was to declare primary and secondary school closures. For adolescents, physical activity (PA) is closely related to school-based activities; pre-COVID-19 pandemic, schools provided between 90 and 150 min per week of PA through physical education and recess. The same closure also affected other activities, such as gyms and various fitness and recreational centers. As a result, during pandemic children typically were engaged in lower levels of PA, fundamental to maintain mental and physical health, and adopted a more sedentary lifestyle as compared to school days [21].

Such necessary restrictions also determined changes in eating habits interfering with the maintenance of a healthy and balanced diet. As a consequence, a greater rate of obesity and nutritional deficiencies has been documented during COVID-19 pandemic [22]. Changes in the nutritional patterns during the pandemic were also due to the financial struggles related to COVID-19 which forced a large number of families to ration food and make cheaper and unhealthy food choices. Furthermore, school closures prevented children belonging to poor families to receive free school lunches and healthy snacks exposing them to food insecurity [23].

This overview aims to describe the consequences of the COVID-19 pandemic on children and adolescents’ lifestyles analyzing the data that has emerged in international literature over the past months.

## Methods

We reviewed the literature analyzing the changes in health-related quality of life of the pediatric population pre and during COVID-19 pandemic to understand the impact of this global health issue on children and adolescents’ lifestyle behaviors. We present results from systematic reviews and meta-analysis, randomized controlled trials (RCTs) and large observational studies published from 2020 so far reporting data about the following issues: changes in eating habits during COVID-19 pandemic; the reduced rate of physical activity in children secondary to schools closures and the limited human activities; the increased rate of obesity among children and adolescents consequent to the sedentary lifestyle and bad eating habits; micronutrients deficiencies related to unhealthy diet; the mental health consequences of quarantine on adolescents leading to sleep disturbances; prevention strategies in terms of nutritional and psychological support among children and adolescents. PubMed has been used as search engine using as key words: COVID-19 and children, SARS-CoV-2 infection and children, COVID-19 and nutrition, COVID-19 and lifestyle.

### The impact of quarantine on children and adolescents

To contain the spread of COVID-19, starting March 2020 states and local governments established restrictions that limited human contact with strong implications in the health, social, and economic fields. In Italy, citizens had to practice social distance maintaining at least 2 m between individuals and avoid social meetings, physical contact, crowded places, and nonessential activities [24]. School closures were also necessary to contain the extension of COVID-19 and forced children and adolescents to stay at home during the lockdown practicing the so-called “distance learning” [20].

The consequences of these confinement measures led to increased sedentary behaviors, with a greater risk of developing or worsening chronic health conditions both on a physical and a mental level even for the young population [25].

Although children and adolescents seem to be less directly affected by the virus, they are struggling against the collateral damages of the COVID-19 pandemic, including inadequate diet, impaired mental health, social isolation, addiction to screens, and lack of schooling and sport activities [26].

#### Changes in food habits

A longitudinal observational study, conducted in Italy, documented negative changes in diet, sleep, and lifestyle behaviors in children and adolescents with obesity during a 3 weeks of home confinement. The study included 41 obese children aged 6–18 years and showed that consumption of potato chip, red meat and sugary drinks increased significantly during the lockdown (*P*-value range: 0.005 to < 0.001) [27].

Another Italian survey conducted from the 5th to the 24th of April 2020, which included 3533 individuals aged between 12 and 86 years, showed that the population group aged between 12 and 17 years have an increased intake of junk food and lower adherence to the Mediterranean diet when compared to the group aged 18–30. In this study population, a higher BMI, as well as a lower age, were associated with increased consumption of packaged sweets and baked products, sweet beverages, savory snacks and dressing sauces [28].

The same results came from a study conducted among 820 adolescents (10–19 years) from Spain, Italy, Brazil, Colombia, and Chile. The reported percentage of adolescents that consumed sweet food every day increased from 14 to 20.7% during confinement. Similarly, the increase of adolescents who consumed fried foods 4–7 days per week, went from 7.4, 3.7, 1.8 and 2.1% to 8.8, 3.8, 2.2 and 2.9% during quarantine [29].

A retrospective cohort study conducted at the Growth Clinic of Seoul St. Mary’s Hospital among school-aged children between 4 and 14 years investigated changes in anthropometric and metabolic parameters during 6 months period of social distancing and school closure due to the COVID-19 pandemic. Data showed an increase in BMI z-scores of 0.219 (95% confidence interval [CI], 0.167–0.271; *P* < 0.001) in the COVID-19 period compared to the 1-year period prior to school closure. Likewise, the proportion of overweight or obesity went from 23.9% in the pre-COVID-19 period to 31.4% in the COVID-19 period [30].

Additionally, 41.7% of the adolescents in Palestine reported weight gain and 50% stated that their food intake increased compared to before the lockdown. Regarding nutrition intake, 31.5% consumed larger quantities of sugar-added drinks (soda, processed juices, etc.), 36.7% reported an increase in fried foods intake, while 46.5% declared a greater consumption of sweets and sugar-added food in comparison to before the lockdown [ 31].

These results confirm that the confinement led to unfavorable eating behaviors and frequent snacking in adolescents considering that food has the power to comfort and support people in stressful conditions [32]. Particularly, foods rich in sugars have a positive effect on mood and reduce stress due to serotonin production [33]. Furthermore, chronic stress activates the hypothalamus-pituitary-adrenal axis, which controls the eventual release of cortisol. Cortisol stimulates highly palatable food intake (e.g., processed foods high in fat and sugar), leading to excessive caloric intake [34].

#### *Physical activities*

Unfortunately, restrictions on human contacts that limit participation to outdoor activities are inevitably disrupting the daily routine of millions of people, including children and adolescents, making it difficult to practice regular physical activity (PA) and exercise [25].

Results that came from various studies show that the current measures are not adequate to maintain sufficient levels of PA at home.

In this regard, a Canadian study provided evidence of collateral consequences of the COVID-19 outbreak in children and youth, demonstrating that only 3.6% of kids (5–11 years) and only 2.6% of adolescents (12–17 years) were practicing 60 min of moderate-vigorous PA/day during the COVID-19 pandemic [35].

Similarly, data derived from an experimental longitudinal study conducted among 2426 children and adolescents (6–17 years) in five schools in Shanghai, showed a drastic decrease in the median time spent in PA which went from 540 min/week (before the pandemic) to 105 min/week (during the pandemic). In conclusion, during the pandemic, the prevalence of physically inactive students increased from 21.3 to 65.6% [36].

Also in Italy children between 6 and 18 years of age with obesity reduced PA to 2.3 (±4.6 SD) hours/week (*P* = 0.003) while screen time increased to 4.8 (±2.4 SD) hours/day (*P* < 0.001) [27].

Each session of moderate-intensity PA promotes the antipathogen activity of macrophages stimulating the recirculation of immune system cells, immunoglobins and anti-inflammatory cytokines in the blood. Thereby, PA can reduce the influx of inflammatory cells in the lungs counteracting pathogen load and attenuating symptomatology of infectious diseases [37].

The downside of the drastic decrease in PA during lockdown, besides its correlation with loss of muscular and cardio-respiratory fitness and weight gain, is that children and adolescents are also experiencing increased levels of anxiety and depression highlighting the positive mental health effects of engaging in physical activity and limiting recreational screen time, especially during stressful periods [36]. On the other hand, increased PA in childhood and adolescence is associated with decreased depressive symptoms [38].

#### Covibesity

Such food habits, in association with a reduction of physical activities, reflect in an increased risk of obesity in children and adolescents as pointed out by the newly developed term “covibesity” which has been introduced to portray the aggravation in obesity rates due to the lockdown imposed during the pandemic [ 39].

According to the *European Society for Clinical Nutrition and Metabolism* (ESPEN), the obesity condition is also related to the severity of COVID-19. It has emerged as one of the most prominent risk factors increasing the disease mortality even in childhood [40]. The pathophysiological process that compromises the functioning of organs and systems in obese individuals includes excessive adipose tissue, deficit in lean mass, insulin resistance, dyslipidemia, arterial hypertension, non-alcoholic steatohepatitis, micronutrients deficiencies, increased oxidative stress, hyperuricemia along with modification of the intestinal microbiota (dysbiosis). These factors lead to chronic subclinical inflammation, impaired immune response, and cardiorespiratory diseases which are the three main risk factors that link obesity to COVID-19. Additionally, obese children are more exposed to the risk of developing severe complications of COVID-19 such as bacterial pneumonia and thromboembolism [41].

Children with obesity have also an increased risk for ventilatory assistance as proved by the fact that in the Children’s Hospital in New York obesity was the most important risk factor for the necessity of respiratory support among 50 pediatric cases of COVID-19 [42].

A recent meta-analysis showed that children who were overweight or obese accounted for 50% (*n* = 136 of 268) of children with MIS-C adding a further possible risk associated with this condition [43].

Interestingly, it has been postulated that childhood obesity rates might increase in proportion to the number of months that schools remain closed: data from the USA estimated that until December 2020 1.27 million new childhood obesity cases were recorded if schools did not reopen [44].

Among the collateral damages of the virus, there are also the economic aspects and their implications in dietary changes considering that many meals for low-income children are provided by schools and childcare centers paying attention to the maintenance of a balanced diet. In fact, because of wide-spread school closures, food insecurity, defined as “having inadequate access to sufficient, safe, and nutritious food to meet dietary needs and food preferences for an active and healthy lifestyle”, had been magnified among low-income children [34, 45].

#### Malnutrition and nutritional deficiencies

Changes in food habits and eating patterns during COVID-19 pandemic, switched to an unbalanced diet, are responsible for an increased risk of obesity and nutritional deficiencies. Infancy and early childhood are key periods for individual growth. Therefore, an unhealthy diet might have a damaging effect on corporeal and weight-for-height development. In addition, since during infancy strongly rooted eating habits are acquired, inadequate nutrition at the pediatric age could have life-long repercussions [46].

Social and income inequalities represent a major determinant for poor outcomes in both developed and developing countries, and COVID-19 pandemic crisis has even more marked these differences. In the middle-and high-income countries the unhealthy diet has favored a high energy density diet and consequently an increased risk of obesity. In contrast, in low-income countries where underweight and overweight could coexist, the balance has moved towards undernutrition [47]. Malnutrition increases morbidity and mortality during infections and causes a significant economic impact on the health care systems [47]. Simultaneously, infections increase the need for several nutrients necessary to maintain bodily integrity and to support immune system activity [48]. In fact, it is well-recognized that nutrition has an important role in modulating the immune response. In this regard, *Calder* et al. have highlighted the importance of optimal nutritional status as a protective factor against viral infections [49]. In applying these considerations to the current COVID-19 pandemic, *Wu and colleagues* have underlined how a nutritional intervention might reduce damages to the lung of patients affected with SARS-CoV-2 [50].

Several vitamins and trace elements, belonging to the group of micronutrients, have been studied in association with the normal functioning of the immune system [51]. Micronutrients regulate enzyme cellular activities, redox processes, and gene expression: consequently, a deficit of one or more of them could partly explain the increased susceptibility to infections and at the same time might offer a potential cue for new preventive and therapeutical approaches based on their supplementations.

**Vitamin A** has been studied for its important function in regulating immune function, in both cellular and humoral immune responses [52]. Studies on infants have shown that Vitamin A supplementation leads to increased production of antibodies after vaccines, like measles [52], anti-rabies vaccination (2.1 times) [53] and influenza virus vaccination, also in children [54]. In order to reduce mortality and risk of complications from pneumonia, croup, and ocular problems it is recommended to correct the low or depleted retinol concentrations resulting from measles infection. This can have some interesting implications considering that severe COVID-19 infection has measles-like manifestations which include fever, cough, and pneumonia but, to date, there is still little evidence to support a beneficial effect of vitamin A supplementation on respiratory infections. However, WHO recommends vitamin A as part of the standard treatment package for all children with acute measles [55].

Similarly, supplementation with **vitamin C** (100–200 mg/day) is known to support respiratory defense mechanisms preventing viral infection which makes it a target of interest in COVID-19 management [56]. In fact, placebo-controlled trials focused on the way to prevent or treat the common cold with oral doses of vitamin C of 200 mg/day demonstrate that the duration of cold episodes is significantly reduced in both children and adults during prophylaxis with an average reduction of 14% in symptom days for children, while in adults the reduction was 8% [57]. Because of its capacity to reduce excessive activation of the immune response, adequate levels of vitamin C are also proved to increase the survival rate of COVID-19 patients. Interacting with both the innate and adaptive immune system, vitamin C encourages antiviral cytokines and free radical formation, hindering viral replication and counteracting abnormal inflammatory responses and hyperactivation of immune cells [58].

Vitamin C modulates the levels of pro-inflammatory cytokines such as TNF-α and promotes the release of interleukin-10 (IL-10) which limits IL-6 levels by a negative feedback mechanism and controls inflammation, critical in COVID-19. In particular, clinical studies suggest that the intake of 1 g/day of vitamin C increases IL-10 secretion by peripheral blood mononuclear cells [59].

**Vitamin D** has been largely studied as a possible protective factor against acute viral respiratory infections suggesting a preventive or even a therapeutic role in COVID-19 [32, 60]. Although vitamin D is usually known for its role in the maintenance of bone health and calcium–phosphorus metabolism, many other functions of this hormone have been recently discovered. In fact, Vitamin D can interact both with the innate immune system, by activating Toll-like receptors (TLRs) or increasing the levels of cathelicidins and β-defensins, and the adaptive immune system, by reducing immunoglobulin and pro-inflammatory cytokines production, thus modulating T cells function and reducing lung inflammation [61–63]. Through human cathelicidin peptide LL37, it also exerts a potent antiviral effect on a variety of viruses, such as HIV-1, influenza viruses, HSV1–2, rhinovirus, and HCV [64–67]. However, it is not known the adequate supplementation regimen to adopt in order to obtain serum levels of vitamin D that guarantee its immunomodulatory role. By the way, serum levels of vitamin D near 20 ng/mL and 50 ng/mL should be enough [67].

The clinical importance of vitamin D within the immune system has been remarkably confirmed during the current pandemic as various metanalysis showed that its deficiency increases the risk of severe COVID-19, hospitalization with COVID-19 and mortality from COVID-19 in adults [68].

Similarly, a study that included 40 patients with COVID-19 aged between 3 months and 18 years and 45 healthy matched control showed that children with COVID-19 had significantly lower vitamin D levels 13.14 μg/L (4.19–69.28) in comparison to the controls 34.81 (3.8–77.42) μg/L (*p* < .001) with fever significantly higher in COVID- 19 patients who had deficient and insufficient vitamin D levels than in patients who had sufficient vitamin D levels (*p* = .038) [69].Despite its correlation with the risk of developing COVID-19 in children, because of the deregulated eating pattern established during pandemic, a significant decrease in 25OHD (18.9 mg/ dL vs. 23.8 mg/dL, *P* < 0.001) was observed among children during the COVID-19 period compared to pre-COVID-19 period pointing out the importance of a vitamin D supplementation during the current pandemic [30].

**Vitamin E** is another fat-soluble vitamin with potent antioxidant action, able to reduce ROS damaging effects [51]. Also in this case, several studies suggested a possible role for vitamin E in modulating immune responses with unknown mechanisms [52]. Moreover, it acts synergistically with selenium in preventing virus-inducing myocardial damage [70], including the one from SARS-CoV-2 [71]. Based on these considerations, vitamin E supplementation might be an option to contain long-term damages of COVID-19. However, in view of the few data available to date, this vitamin is not used as a preventive strategy for viral infections.

The **iron** role is controversial. It is a trace element influencing the number and activity of T CD4 circulating cells, facilitating a higher Th1 subtype response than Th2 [72–74]. In fact, it has been reported a predominance of allergy in children and adolescents with iron deficiency and an elevated risk to develop atopic diseases in children whose mothers have had low iron levels during pregnancy [75, 76]. However, this element is also a source of nourishment for many pathogens [77]. Not surprisingly anemia is one of the clinical and laboratory during an inflammatory chronic process aimed to reduce the substrates for pathogens [78]. Therefore, it is necessary for a good iron plasmatic homeostasis to balance its pro-inflammatory and anti-inflammatory effects. A preliminary evaluation of serum iron, ferritin and transferrin should be made before considering any possible supplementation.

In contrast, the use of **zinc** compounds might be an adjunct therapy in COVID-19 treatment. Beyond its ability to modulate the immune system, zinc can improve barrier functions enhancing cilia’s morphology and increasing its length and beating frequency [79]. Moreover, it promotes antiviral and antioxidants actions that offer protection to the respiratory epithelial lining. For these reasons, an uptake of up to 40 mg per day of zinc is recommended for adults to reduce the potential threat of the COVID-19 pandemic, resulting from the rise in the host resistance to viral infections [80]. Indeed, a study conducted in Thailand among children aged between 2 and 60 months found that acute lower respiratory tract infections solved faster in the ones who received zinc supplementation (median (IQR): 3 (2–4) days and 4 (3–5) days, respectively; *P* = 0.008), and that their hospital stay was shorter (mean (SD): 3.8 (1.3) days and 6.1 (3.2) days) than the placebo group [81]. Based on these considerations, it could be assumed that zinc supplementation may be useful also for children.

Likewise, **selenium** (Se) is an essential trace element relevant for a well-balanced immune response [82]. In fact, a cross-sectional study of patients affected by COVID-19 conducted in Germany demonstrated that Se status analysis in COVID patients provides diagnostic information for a better prediction of the disease course and of the risks associated. A Se status below < 45.7 μg/L was documented in 43.4% of COVID samples in contrast with the levels of selenium found in samples from surviving COVID patients which were significantly higher in comparison to non-survivors (Se; 53.3 ± 16.2 vs. 40.8 ± 8.1 μg/L) suggesting a possible role of this trace element in facing the viral infection. In light of these results, Se supplementation could represent an adjuvant therapy in COVID-19 patients [83]. Furthermore, an interesting correlation between COVID-19 cure rate and city population selenium status based on hair selenium concentration was found in 17 cities outside Hubei, China [84].

**Hypomagnesemia** could represent another factor worsening the prognosis of patients affected with SARS-CoV-2 infection. It has been shown a direct correlation between low levels of Magnesium (Mg) and different metabolic disease and persistent chronic inflammation such as hypertension, metabolic syndrome, Type II Diabetes, cardiovascular diseases, osteoporosis and malignant tumors, typically associated with a severe course of COVID-19 disease [85, 86]. Conversely, an oral supplementation of Mg has been associated with reduced levels of C-reactive protein (PCR) [87]. Therefore, with the ongoing COVID-19 pandemic, oral Mg supplementation could be a potential prevention strategy thanks to its ability to reduce blood pressure and to mediate an antithrombotic effect [88]. As confirmation, the mortality associated with COVID-19 disease is reset in patients with normal Mg blood levels and its supplementation reduces the need for oxygen therapy and the ICU hospitalization for patients affected with SARS-CoV-2 infection [89, 90].

Finally, long-chain **omega-3 fatty acids** (FAs), the main components of fish oil which include eicosapentaenoic (EPA) and docosahexaenoic (DHA) FAs, are also well known to have beneficial effects on immunity and inflammation. According to *Gutierrez* et al. omega-3 FAs are incorporated into the cell membrane of neutrophils where they can be metabolized into prostaglandins, leukotrienes, thromboxanes, maresins, protectins, and resolvins which then modulate neutrophil migration, phagocytic capacity, as well as the production of reactive oxygen species and cytokines. Moreover, omega-3 FAs improve the function of the macrophages promoting their phagocytic capacity and down-regulate Nuclear Factor-κ Beta (NFκB), which in turn reduces inflammatory markers like IL-6, TNFα, and tissue growth factor-beta [91]. By reducing the release of proinflammatory mediators through resolvins and other metabolites, EPA and DHA interfere with pulmonary neutrophils recruitment, increase apoptosis by macrophages, and subsequently decrease broncho-alveolar IL-6 production and as a result, turning off the inflammation of the lung and preventing the cytokine storm. Interestingly, omega-3 FAs also exert anti-viral effects by inducing interferon (IFN) which inhibits viral replication [92]. Based on these considerations, *the European Society for Parenteral and Enteral Nutrition* supports the use of omega-3 FAs to ameliorate complications of COVID-19, although firm evidence is still missing [93].

In addition to micronutrients, the scientific society has evaluated the potential beneficial effects of several herbals and probiotics in reducing the morbidity and mortality COVID-19 associated [94]. It has been suggested that plants could offer protection against viral infection, through polyphenols, flavonoids and isocyanates such as turmeric, quercetin, resveratrol and, sulforaphane [95, 96]. Based on this evidence, since the beginning of the COVID-19 pandemic, Chinese doctors have proposed the employment of these substances to offer protection against the harmful effects of SARS-CoV-2 virus [97]. By exploiting the inhibitor effect in vitro against MERS virus, **resveratrol** has been studied also for SARS-CoV-2 infection [98]. It is a polyphenolic compound, chemically known as *trans*-3,5,4′-trihydroxystilbene, that can be found in red grapes, berries, peanuts, and bamboo [99]. Besides its ability to reduce virus replication, the anti-oxidative and anti-inflammatory effect [100], this polyphenol forms an extremely reliable compound with the human ACE-2 transmitter inhibiting virus interaction with target cells [101]. Furthermore, it is able also to activate SIRT1 and p53 signaling pathways and increase cytotoxic T lymphocytes (CTLs) and natural killer (NK) immune cells [102]. Although there is no indication to use resveratrol to treat SARS-CoV-2 infection, it may be a beneficial adjunctive antiviral agent to consider.

Similar to resveratrol, **curcuma** (a therapeutic rhizomatous plant of the *Zingiberaceae* family known as turmeric) can limit the virus’s intracellular penetration binding on its surface. Add to this, it reduces the cytokine storm by lowering the NFkB production and, blocking the production of TNF-α, it stops the fibrotic degeneration of lung, kidneys, cardiac muscle cells and hematopoietic system cells [103]. Its potential antidepressant and anti-anxiety effect should be not underestimated [104]. **Quercetin** is a flavonoid that is the most abundant in vegetables and fruits [105]. It acts modulating gene expression of 98/332 human genes (30%) codifying for cellular receptor proteins for SARS-CoV-2 virus [106]. *Abian* et al analyzing a small chemical library of about 150 compounds, identified the quercetin, a potent inhibitor of SARS-CoV-2 3CLpro (K_i_ ~ 7 μM) [107]. 3CLpro is a potent viral protease necessary for SARS-CoV2 replication. Biophysical techniques and binding to the active site in molecular simulations have allowed to know the exact mechanism with which quercetin interacts with 3CLpro, making it a candidate for COVID-19 therapeutic treatment. In this regard, it is underway a randomized, open-labeled, and controlled study aimed to investigate the adjuvant benefits of quercetin phytosomes in community-based subjects with confirmed SARS-CoV-2 infection (by RT-PCR). The study has two arms: in the first arm, there are subjects receiving standard COVID-19 care per the hospital/physician guidelines, whereas in the other, the subjects are treated with routine COVID-19 care and quercetin phytosomes. It is proposed that quercetin phytosomes, after 30 days of supplementation, will boost the natural immunity of the subjects and help to prevent the COVID-19 disease progression, reducing the need for hospitalization [108]. Finally, **sulforaphane**, a natural activator of nuclear factor erythroid-2-related factor 2 (Nrf2) and the most powerful human antioxidant, has been suggested as preventive strategy for severe COVID-19. In fact, SARS-CoV-2 virus determines ACE2 destruction after being linked to it, resulting in angiotensin II receptor type 1 (AT1-R) activation with consequent oxidative stress, insulin resistance and endothelial lung damage [109]. The first observations derived from the finding of a low mortality in East Asia, central Europe and Balkans, where there is a high consumption of fermented foods and cabbage, rich in sulforaphane [110]. Therefore, his assumption through cabbage and broccoli could offer protection against the severe forms of disease.

Over the last decades the scientific interest has been focused on understanding the effective role of probiotics as a preventive and therapeutical measure for respiratory infections, including COVID-19 disease [111, 112]. Probiotics, such as *Lactobacillus* and *Bifidobacterium* bacteria, are nonpathogenic living microorganisms, typically present in fermented food. Several studies have observed a reduction of viral infections and a mild course of the disease in patients treated with them [113]. In addition, they have also been successfully used both for the treatment and the prevention of gastrointestinal illnesses and inflammatory processes of allergic nature [114, 115].

Regarding COVID-19, experiences with other viral infections, such as influenza, rhinovirus, and respiratory syncytial virus, have shown some beneficial effects shaped to conclude that probiotic supplementation can be useful for the prevention of SARS-CoV-2 infection [116]. As the infectious agent is transmitted also through feces, particularly in children, the administration of probiotics could interfere with this mechanism reinforcing the gut epithelial barrier and directly competing with the proliferation of SARS-CoV-2 [117].

Furthermore, it seems that in patients treated with probiotics there is a reduction of viral transmission due to the enhanced local and systemic immune response and the creation of a “gut-lung axis” which finally favors the clearance of the infectious agent and consequently reduced contagiousness [118].

Although there is no specific evidence on the strain of probiotics to be used, the posology and the duration of the intervention, especially in children, most of the studies on the prevention of pediatric respiratory infections are focused on the effects of *Lactobacillus* strains and *Bifidobacterium* strains (or mixtures of strains), usually administered for 3 to 12 months [119].

Nevertheless, further studies are needed to better define their potential use as an adjuvant therapy against viral infections, including SARS-CoV-2.

#### Mental health and sleep disturbances

Prolonged quarantine, fear of infection, frustration and boredom, the lack of contact with classmates and teachers and the lack of space at home are just some of the factors that are causing psychological repercussions in children and adolescents during the pandemic [120].

COVID-19 related quarantine implied monotony, stress, impatience, annoyance and varied neuropsychiatric manifestations. In this regard, the 2020 National Report on medicines use in Italy, prepared by the *Italian Medicines Agency* (AIFA) and the *Medicines Utilization Monitoring Centre* (OsMed), documented an increase of 11.6% in using of psychiatric drugs in the pediatric population compared to the previous year. Particularly, antipsychotics are the drugs that recorded the greatest increase in prescriptions (+ 17.2%) followed by antidepressants and medications used for Attention deficit-hyperactivity disorder (ADHD) such as methylphenidate and atomoxetine [121].

In a survey among 8079 Chinese adolescents aged 12–18, *Zhou* et al. reported a high prevalence of symptoms of depression (43%), anxiety (37%) and mixed anxiety and depression (31%) during the COVID-19 outbreak [122]. In this regard, online psychotherapy can help in providing support to patients with anxiety and stress-related disorders spreading online resources such as information about mental health education, video-counseling, telemedicine and telepsychiatry services. In particular, cognitive-behavioral therapy (CBT) and mindfulness-based cognitive therapy (MCBT) have a strong impact on reducing stress and maladaptive coping behaviors [123].

Sleep disturbances that occurred during SARS-CoV-2 pandemic can also have a major impact on emotional health and immune function. Insufficient sleep increases cardiometabolic disease risk in both children and adolescents and results in anxiety or mood swings, which may be exacerbated by poor mental health during the COVID-19 pandemic [25]. On the other hand, excessive sleep, as documented by *Pietrobelli* et al. with an increase of 0.65 h per day during lockdown among Italian children compared to data of 2019, could also alter the circadian system [27]. Additionally, the increase of screen time has a negative influence on sleep balance considering that blue light exposure from device screens near bedtime can suppress melatonin release [124]. Given this association, the American Academy of Pediatrics recommends avoiding screens at least 1 h before bedtime with specific limits on screen time equal to 1–2 h per day [125, 126].

However, during pandemic, *Alves* et al., reported an average of 6 h a day of leisure screen time among children aged from 9 to 15 years [34]. Same results came from *Pietrobelli* et al. who documented an increase in screen time of 4.85 h per day (*P* < 0.001) [39].

Besides, during social isolation, the intensive use of the internet and social media can spread the practice of the so-called online “challenges” that often lead to self-harm and suicidal intentions in youth [127].

#### Prevention strategies

**Fig. 1:**
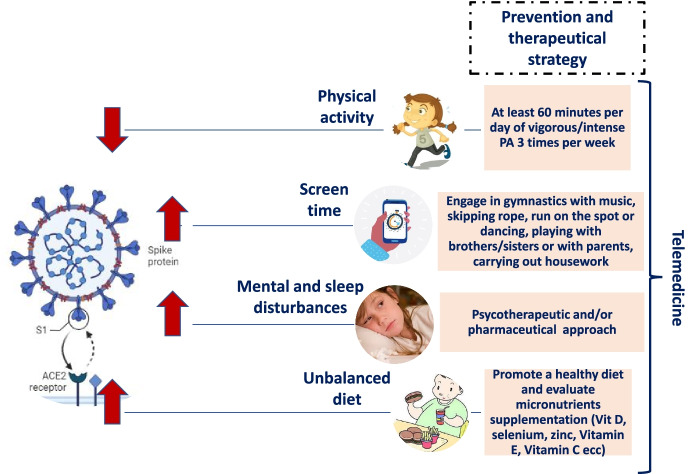
The impact of the SARS-CoV-2 infection on children and adolescents. During the actual pandemic the pediatric population has been involved in severe negative changes in diet, sleep quality and lifestyle behaviors resulting in a greater rate of sedentarity and mental disturbances. Therefore to contain the spread of Covibesity it is recommended to increase physical activities, have a balanced diet and simoultaneously, take into account the possibility of a psycological support

Currently, there is no preventive and curative treatment accepted for SARS-CoV-2 infection. There has been a great effort in seeking drugs able to interfere with viral replication or to stop the progression towards a clinical severe form of the disease, with poor results. In this regard, the WHO research forum on COVID-19 has recently published in ‘*New England Journal Medicine*’ data about the use of repurposed antiviral drugs (remdesivir, hydroxychloroquine, lopinavir, and interferon beta-1a) for SARS-CoV-2 infection [128]. The study has been conducted in 11,330 adults hospitalized with COVID-19, by engaging 405 hospitals of 30 countries worldwide. Results obtained have not documented any difference in reducing the duration of hospital stay, the need for assisted ventilation and, mortality by comparing patients treated with trial drugs with those treated with standard of care. In the pediatric population data are even less, not allowing the achievement of a universal therapeutic intervention plan. Currently, treatment in children infected with SARS-CoV-2 consists mainly of supportive care, including oxygen and advanced respiratory support, hydration, and antipyretics. Since a relevant viral load, an immune response uncontrolled and pre-existing clinical conditions are physiopathological factors influencing the clinical course of SARS-CoV-2 infection, both control of viral replication and immunomodulation might help to deal with harmful effects of viral infection. Hence the idea to find alternative approaches for new potential treatment strategies [128] Fig. 1.

In this regard, during COVID-19 pandemic a good option to promote more balanced dietary habits and healthy lifestyles is represented by telemedicine, defined by the Institute of Medicine as the use of electronic and telecommunications technologies to provide and support healthcare when distance separates the participants [129]. In fact, the efficacy of computer-based cognitive-behavioral treatments (cCBT) in children and adolescents with anxiety and depression had already been provided by *Ebert* et al. [130] One of the greater advantages of Internet-based interventions (IMIs) is the possibility to easily include them into the daily activities of the patients. They can help people to overcome the fear of stigmatization by preserving anonymity and allowing individuals to be more aware of their personal progress [131]. Furthermore, Telehealth represents a valid option for treating children with obesity as already confirmed by the decrease in BMI documented by *Fleischman* et al. after 3 months of primary care provider’s visits integrated with telematic assistance [132]. Virtual consultation in the nutrition practice is feasible and encouraged during the COVID-19 pandemic because it has the potential to improve the care provided by practicing registered dietitian nutritionists reducing no-show rates and increasing retention as well as improving health outcomes for patients [133].

Several apps with personalized training features have also emerged offering motivation to people in reinforcing their health by practicing more PA. In conclusion, the pandemic is forcing the health system to find alternatives to face-to-face consultation. In this context, a valid option is represented by telemedicine which allows people to access medical and psychosocial assistance during COVID-19 pandemic in an easier way.

#### Nutritional approach in the prevention of SARS-CoV-2 infection

In relation to dietary factors and food habits, WHO has proposed a set of general suggestions such as limiting the consumption of salt, sugar, fat, and alcohol and encouraging the consumption of fiber, water, and fresh products, especially fruits, vegetables, and reduced-fat dairy [134]. Similarly, the *Italian National Institute of Health* suggests healthy and balanced nutrition based on a reduced intake of fats, carbohydrates and sugary drinks. Moreover it suggests, taking advantage of forced quarantine, having a balanced diet for all family members [135].

As previously described, there is a strong relationship between the immune system and nutrition. An adequate nutritional intake is necessary to provide a supply of energy and nutrient for the maintenance and replication of the immune system cells. Simultaneously, the detrimental effect of malnutrition on determining resistance to infections have been described [49, 136].

Most of the current therapeutical effort is aimed to inhibit viral replications and to regulate the “cytokine storm”, conditions characterized by elevated production of proinflammatory mediators [cytokines Interleukin (IL)-6, tumor necrosis factor (TNF)-alfa, IL-8] [137], resulting from the oxidative stress state related to the virus replications and the consequent general inflammatory state. Moreover, SARS-CoV-2 virus itself may interfere with the equilibrium between NFkB transcription molecules involved in the expression of cytokines and Nrf2 activation, responsible for the expression of antioxidant enzymes [113]. Hence, the need to find therapies able to interfere with this pathophysiological phase of the disease limiting the deleterious effect of SARS-CoV-2 virus on lungs and the rest of the organs.

Lately, there has been a growing interest of the scientific society in nutraceuticals, natural substances with beneficial effects on health. Although it is rare to find them in natural foods in sufficient quantities to obtain these benefits [138], they can be extracted and used as food supplements or additives.

In the pediatric field, the term nutraceutical is used to refer to adequate diet enriched with biologically active elements allowing to obtain health benefits beyond the normal nutritional effects [139, 140]. Therefore, it is possible to define them as functional foods rich in vitamins, mineral salts, fibers, and fatty acids in such quantities as to positively influence body functions. To date, two classes of nutraceuticals have been described: those that contribute to adequate body growth and those that offer protection against infections [141, 142]. Over time, a lot of nutraceuticals have been proposed. However, the link between the intake of a certain substance, such as vitamins, and the prevention of infections is not always demonstrated, so the possibility to use nutraceuticals to prevent infections, especially respiratory infections, remains questioned. This concept becomes even more important considering the ongoing COVID-19 pandemic. Basic hygienic practices, proper dietary and lifestyle behaviors are necessary to prevent respiratory viral diseases. The meals distribution over the day should provide at least five portions of fruit and vegetables each day and all main meals should contain a balanced proportion of macronutrients to achieve an adequate caloric daily intake [143].

Adequate levels of vitamins and micronutrients are fundamental to improve immune responses as they have antiviral effects and may reduce the severity of symptoms and the duration of respiratory infections. Therefore, an adequate dietary intake of these substances is crucial to ensure the protective effects of the immune system. Fruits, vegetables, meat, fish, poultry and dairy products are the main source of these vitamins and minerals. In particular, vitamin D can be found in anchovies, swordfish, tuna, salmon but also mushrooms and egg yolk. Similarly, fruits (i.e. orange, grapefruit, lime, mandarin, strawberries, kiwi, pineapple), tomato, broccoli, cabbage and spinach, are nourishments rich in vitamin C. Vitamin E instead is found in dried fruits (hazelnut, almonds, peanuts), vegetable oils, green leafy vegetables such as chicory, mango, avocado and fish products like salmon and shrimps. Zinc and selenium can be found in mature cheese, nuts (especially cashews and pecans), both red and white meat and fish products like lobsters, mussels, clams and mullets. Lastly, it should be remembered that fish products, especially salmon, herring, mackerel, sea bass and sea bream are the main source of long-chain omega-3 FAs [59, 144, 145]. In particular, EPA occurs in large quantities in herring, wild sardine and pollock roe whereas in plants it is rather rare; on the other hand, the main sources of DHA are flying fish, herring, pollock and salmon roe [146].

However, considering that for several logistic and financial difficulties it is difficult to maintain a balanced diet during lockdowns or self-quarantine, multi-vitaminmineral (MVM) supplementation for a short period during this COVID-19 pandemic might have a beneficial effect. Indeed, even more attention should be paid to subjects who are malnourished or at risk of malnutrition with the aim to offer them protection against the viral infection and its consequences [147].

Based on the patient’s risks factors and baseline 25OHD levels, a daily vitamin D supplementation with 400–1200 UI has been suggested for pediatric patients ^154.^ Therefore, a supplementation within this posology range can reasonably be suggested for the prevention of COVID-19 considering the countless benefits that this would also have in supporting the growth and development of the individual, regardless of the current pandemic [148].

Regarding supplementation with high doses of vitamin C and E, there is no sufficient evidence about the beneficial effects of such intervention during viral infections, including SARS-CoV-2. Therefore, at the current time, vitamin C and E supplementation is not strictly recommended [57, 149].

Although less common in pediatric patients, it has been evidenced that 20% of the general global population, especially elderly patients, or individuals affected by chronic cardiac or pulmonary disease, hypertension and diabetes, have a low zinc intake. Based on these considerations, a daily dose of 10–20 mg of zinc for 6–12 months is reasonable to suggest to help in the prevention of COVID-19 [43, 148]. Similarly, recent studies support selenium supplementation (50 mg/daily) [150]. In Table 1 the suggested posology and the food-sources of nutritional supplements in the prevention of COVID-19 are summarized.

**Table 1:** Nutritional supplements in the prevention of COVID-19 disease

Nutritional supplement	Suggested posology	Food sources
Vitamin A	50,000–200,000 IU every 4–6 months	Cheese, eggs, oil fish, fortified low-fat spreads, milk and yogurt
Vitamin C	100–200 mg/daily	Fruits (i.e. orange, grapefruit, lime, mandarin, strawberries, kiwi, pineapple), tomato, broccoli, cabbage and spinach
Vitamin D	400–12,000 IU/daily	Anchovies, swordfish, tuna, salmon, mushrooms and egg yolk
Vitamin E	6–11 mg/daily^a^	Dried fruits (hazelnut, almonds, peanuts), vegetable oils, green leafy vegetables such as chicory, mango, avocado, and fish products such as salmon and shrimps
Iron	7–13 mg/daily^a^	Red meat, beans, nuts, dried fruit such as dried apricots, fortified breakfast cereals and soy bean flour
Zinc	10–20 mg/daily	Mature cheese, nuts (especially cashews and pecans), both red and white meat and fish products such as lobsters, mussles, clams and mullets
Selenium	50 mg/daily	
Magnesium	170–250 mg/daily^a^	Dark chocolate, avocado, nuts, legumes, tofu, seeds, whole grains, fatty fish (salmon, mackerel and halibut) and bananas
Long chain omega-3 fatty acids (FAs)	100–250 mg/daily^a^	Fish especially salmon, herring, mackerel, sea bass and sea bream

^a^ European Food Safety Authority, available at www.efsa.europe.eu

Currently, there is little evidence about the use of nutraceuticals to prevent viral infections. However, considering the non-toxicity of nutraceuticals, they could be used to potentiate immunity. Although different products have been proposed (garlic, oily fish, cranberry juices and broccoli sprouts), it is difficult to find them and for this reason, their use is actually limited [151–153].

In conclusion, it is important a nutrition assessment for patients affected with COVID-19 evaluating the dietary habits and the possible need to receive nutrition support, including vitamin supplementations.

#### Physical activity promotion

Based on guidelines issued by the WHO, the *Italian National Institute of Health* suggested recommended levels of PA for different age groups, including adolescents aged 12–17 years during the COVID-19 outbreak emergency [154]. They propose at least 60 min per day of vigorous/intense PA 3 times per week including exercises that strengthen muscles and bones. It doesn’t matter the place where PA is practiced, even at home, with no special equipment and with limited space. Children and adolescents should engage in gymnastics with music to keep conditional skills trained (strength, endurance, speed) and stimulate cardiovascular and respiratory systems, skipping rope, run on the spot or even some suitable transversal activities like dancing, playing with brothers/sisters or with parents, carrying out housework (dusting, vacuuming, setting/clearing the table, making their bed) [155].

#### Vaccination

The only strategy which allows taking a stop to the SARS-CoV-2 spread is vaccination. To September 2021, a total of 671,767,335 COVID-19 vaccine doses have been distributed by manufacturers to EU/EEA countries of which Comirnaty (BNT162b2), developed by BioNTech/Pfizer, represents 69% of all doses distributed to EU/EEA countries via the European Commission’s Vaccine Strategy, followed by Vaxzevria (AZD1222) (14%), Spikevax (12%), and COVID-19 Vaccine Janssen (3%). Based on data available from 29 countries, 85% of the doses distributed in the EU/EEA since the beginning of the rollout have been administered.

**S**cientific data currently available on vaccination for children and adolescents are poor. In fact, it is not yet clear if SARS-CoV-2 vaccine could ensure prolonged immunogenicity or potential adverse effects in children. However, adverse effects have been reported only in 5 out of 1131 vaccinated children, and none of these were related to the study intervention [156, 157].

Data on long-term effects are not yet available, and rare age-specific undesirable effects may only emerge with increasing vaccination. The only accepted adverse effect is a possible appearance of SARS-CoV-2 myocarditis in vaccinated young patients [158–160], event thought the incidence reported is currently low [161].

Despite the lack of extensive data on the real effectiveness of vaccination against SARS-CoV-2 in children and adolescents, these vaccinations appear to be safe [156, 157]. Therefore, the main scientific societies are in accord to extend the SARS-CoV-2 vaccine administration also to pediatric patients. Until recently all children over 12 years of age have received vaccination with mRNA vaccines (BioNTech/Pfizer or Moderna). On November 25th EMA’s human medicines committee (CHMP) has recommended granting an extension of indication for the COVID-19 vaccine Comirnaty to children aged 5 to 11 after evidence declaring the superiority of benefits over risks in this age group. Starting in December 2021, the consensual approval of the Italian Medicines Agency (AIFA) has allowed the extension of vaccination practice to Italian children aged 5–11 years with a lower dose of Comirnaty that used in people aged 12 and above (10 μg compared with 30 μg). As in the older age group, it is given as two injections in the muscles of the upper arm, three weeks apart. This is only the first step who will guide towards overcoming the SARS-CoV2 global pandemic.

## Conclusions

The COVID-19 pandemic has a major impact on health and lifestyle behaviors. Children, although less directly affected by the virus, are paying a heavy price through the indirect effects of the crisis, including unbalanced diet leading to an increased risk of both overweight and underweight, sedentary lifestyle, mental health impact and social isolation, addiction to screens and lack of schooling and health care. The COVID-19 quarantine is the only way to prevent virus dissemination, especially in children. However, studies report adverse effects on psychological well-being such as anxiety, worrying, irritability, depressive symptoms, and posttraumatic stress disorder symptoms among children worldwide [162].

Apart from the negative aspects of the current pandemic, facing the consequences of the COVID-19 crisis together with family members could also provide the opportunity to strengthen the sense of community and cohesion inside them promoting social support and resilience. Moreover, among the beneficial consequences of the current crisis, home-schooling could have helped children who have experienced bullying or other stressors during school time. Overcoming the trauma linked to the current pandemic could promote personal growth and psychological development which in turn reinforce self-confidence and become a protective factor for coping with future stressors [163].

As a plan of a comprehensive global intervention, social protection programs, public awareness campaigns and nutrition education programs should not be missing, especially for precarious groups. Pediatricians should actively give prevention information educating their patients and families. At the same time, more attention should be paid to poor families by providing them the economical financial support necessary to guarantee a good quality of life, especially from a nutrition standpoint.

Regarding prevention strategies, scientific attention is focusing on seeking alternative prevention approaches that, beyond the well-known rules aimed at reducing the spread of the virus (masks, social distancing), offer the possibility of preventing damaging effects on the children and adolescents’ health. In this regard, the starting point could be a food education intervention aimed to ensure a balanced eating regimen in terms of micro- and macronutrients. Add to this, the use of nutraceuticals, known to be effective in protecting against respiratory viruses, might be a strategical therapeutical approach to prevent SARS-CoV-2 related consequences.

Considering this evidence, although there is no adequate scientific consensus about the possibility to use nutritional support as prevention measure against respiratory infections of children and adolescents [164], the current scientific knowledge should be pushed towards a more careful analysis in this field to get advantages out for the management of COVID-19 pandemic. However, given the relative safety of these substances, their use could still be suggested, especially in children.

The only way to stop the current COVID-19 pandemic is to inhibit the virus spread through vaccination that currently is possible over 12 years of life. Fortunately, further pediatric clinical studies are underway intending to extend the SARS-CoV-2 vaccination to the whole pediatric population.
